# Two-Component Nanoparticle Vaccine Displaying Glycosylated Spike S1 Domain Induces Neutralizing Antibody Response against SARS-CoV-2 Variants

**DOI:** 10.1128/mBio.01813-21

**Published:** 2021-10-12

**Authors:** Linda van Oosten, Jort J. Altenburg, Cyrielle Fougeroux, Corinne Geertsema, Fred van den End, Wendy A. C. Evers, Adrie H. Westphal, Simon Lindhoud, Willy van den Berg, Daan C. Swarts, Laurens Deurhof, Andreas Suhrbier, Thuy T. Le, Shessy Torres Morales, Sebenzile K. Myeni, Marjolein Kikkert, Adam F. Sander, Willem Adriaan de Jongh, Robert Dagil, Morten A. Nielsen, Ali Salanti, Max Søgaard, Timo M. P. Keijzer, Dolf Weijers, Michel H. M. Eppink, René H. Wijffels, Monique M. van Oers, Dirk E. Martens, Gorben P. Pijlman

**Affiliations:** a Laboratory of Virology, Wageningen University, Wageningen, The Netherlands; b Bioprocess Engineering, Wageningen University, Wageningen, The Netherlands; c AdaptVac Aps, Hørsholm, Denmark; d Laboratory of Biochemistry, Wageningen University, Wageningen, the Netherlands; e Laboratory of Phytopathology, Wageningen University, Wageningen, The Netherlands; f QIMR Berghofer Medical Research Institute, Brisbane, Australia; g Department of Medical Microbiology, Leiden University Medical Center, Leiden, Netherlands; h Centre for Medical Parasitology at Department for Immunology and Microbiology, Faculty of Health and Medical Sciences, University of Copenhagen, Copenhagen, Denmark; i ExpreS2ion Biotechnologies Aps, Hørsholm, Denmark; j Applikon Biotechnology BV, Delft, The Netherlands; Indiana University Bloomington

**Keywords:** SARS-CoV-2, insect cells, nanoparticle, vaccines

## Abstract

Vaccines pave the way out of the SARS-CoV-2 pandemic. Besides mRNA and adenoviral vector vaccines, effective protein-based vaccines are needed for immunization against current and emerging variants. We have developed a virus-like particle (VLP)-based vaccine using the baculovirus-insect cell expression system, a robust production platform known for its scalability, low cost, and safety. Baculoviruses were constructed encoding SARS-CoV-2 spike proteins: full-length S, stabilized secreted S, or the S1 domain. Since subunit S only partially protected mice from SARS-CoV-2 challenge, we produced S1 for conjugation to bacteriophage AP205 VLP nanoparticles using tag/catcher technology. The S1 yield in an insect-cell bioreactor was ∼11 mg/liter, and authentic protein folding, efficient glycosylation, partial trimerization, and ACE2 receptor binding was confirmed. Prime-boost immunization of mice with 0.5 μg S1-VLPs showed potent neutralizing antibody responses against Wuhan and UK/B.1.1.7 SARS-CoV-2 variants. This two-component nanoparticle vaccine can now be further developed to help alleviate the burden of COVID-19.

## INTRODUCTION

Vaccination has become a key instrument in the fight against the severe acute respiratory syndrome coronavirus 2 (SARS-CoV-2) outbreak, which was declared a pandemic by the World Health Organization in March 2020. Within 6 months, the coronavirus disease 19 (COVID-19) had claimed the lives of one million people (https://covid19.who.int). Despite global efforts to restrict the viral spread through economic and social interventions, the virus continues to put a substantial strain on economies and health care systems around the world. Large-scale vaccination programs have proven to be critical in reducing the viral spread and preventing severe disease (1).

The envelope of the SARS-CoV-2 virion contains membrane and spike (S) proteins. The S protein is a trimeric glycoprotein involved in virion attachment and entry into host cells. S is divided into two domains, S1 and S2, by a furin protease cleavage site (2, 3). S1 contains the receptor-binding domain (RBD) that binds the human angiotensin 2 (hACE2) receptor, whereas the fusion peptide (FP) is found in S2 (4, 5). Since S is indispensable for virus entry and is highly immunogenic, it is the main target in vaccine design to induce antibody-mediated virus neutralization in immunized individuals (6, 7). In many vaccine development studies, S is stabilized in its prefusion state by eliminating the furin cleavage site and inserting a stabilizing diproline mutation in S2 (3, 8–10).

At unprecedented speed, multiple COVID-19 vaccines have entered the market via emergency approvals from, among others, the European Medicines Agency and the U.S. Food and Drug Administration. These early vaccines, which are based on mRNA or adenoviral vectors, have been shown to be effective in preventing COVID-19 infection (11–13). Recombinant subunit vaccines based on recombinant S protein are currently in late-stage clinical trials and have been shown to induce potent neutralizing antibody (nAb) responses in nonhuman primates (14–16) and humans in phase II and III clinical trials (17).

The recent emergence of SARS-CoV-2 variants (https://nextstrain.org/sars-cov-2) highlights the importance of a robust vaccine production platform with good scalability and modularity for rapid adaptation to novel variants. For the production of recombinant proteins, the insect cell-baculovirus expression vector system (IC-BEVS) is a well-established platform. It is used for (commercial) producing virus-like particles (VLPs; human papillomavirus; Cervarix, GlaxoSmithKline), proteins with complex posttranslational modifications, including glycoproteins (influenza A virus hemagglutinin; FluBlok; Sanofi), and difficult-to-produce protein complexes (18, 19). IC-BEVS has been used to produce coronavirus spike proteins of SARS-CoV (20) and Middle East respiratory syndrome-coronavirus (MERS-CoV) (9, 21, 22) as well as SARS-CoV-2 spikes in structural studies (23–27) and as subunit vaccines (28, 29). The IC-BEVS is rapidly scalable up to a million doses using insect cell bioreactors with working volumes of a few thousand liters in animal component-free medium (18, 30, 31). Furthermore, protein-based vaccines offer a very high safety profile, since millions of people have been vaccinated with IC-BEVS-produced proteins (Cervarix, FluBlok) without experiencing any severe adverse effects (31, 32). Therefore, IC-BEVS would be well-suited for producing SARS-CoV-2 vaccines.

A potential shortcoming for such subunit vaccines is their relatively low immunogenicity that can be compensated for by the addition of adjuvants and/or sequential immunizations (boosters). This has also been demonstrated in the case of SARS-CoV-2 RBD immunization (33, 34) and prefusion stabilized S (35). The immunogenicity of subunit vaccines can be improved by the presentation of antigens on nanoparticles or VLPs. The unidirectional display of antigens and the size of the particles more closely resemble that of a virus and result in increased B-cell responses by cross-presentation of epitopes, resulting in enhanced processing by antigen-presenting cells (APC) and B-cell receptors (36–38). Moreover, VLPs are replication-deficient because they do not contain genetic material from the originating virus and, thus, are considered safe (39). The licensed human papillomavirus vaccine (Cervarix; GSK) is a VLP made with the IC-BEVS that provides long-lived protective immunity after just one dose (40, 41). Experimental SARS-CoV-2 VLP and nanoparticle vaccines have also been developed and have demonstrated their potential to induce protection against disease with low doses (14, 42–44).

In this study, we use the AP205 VLP and tag/catcher system to construct a baculovirus-derived SARS-CoV-2 vaccine based on the S1 subunit. Covalent conjugation of antigens on the VLP surface can be achieved by the Spytag/Spycatcher platform (45, 46). This plug-and-display technology is based on a bacterial adhesin that has been split genetically into a tag and catcher peptide, which forms a covalent bond upon mixing (47). Even without adjuvant, AP205 VLPs decorated with antigens have been found to induce robust immune responses at a single dose (48, 49). Recently, the RBD domain of SARS-COV-2 was coupled to these VLPs using a proprietary split-protein tag/catcher technology, resulting in robust neutralizing antibody production in mice (34), and has been further developed into a phase I/II clinical study.

Using the IC-BEVS, we demonstrate insect cell-mediated production and purification of (i) the full-length spike protein, (ii) a secreted form of a prefusion-stabilized spike, and (iii) a secreted form of the SARS-CoV-2 S1 domain. We formulated the secreted S as an adjuvanted subunit vaccine and tested protection of K18-hACE2 mice from SARS-CoV-2 challenge. We also fused the S1 domain to the split-protein tag and displayed this on AP205 VLPs. Mice vaccinated with the S1-VLP vaccine had strong immune responses and neutralizing antibodies against both the Wuhan SARS-CoV-2 and the UK/B.1.1.7 variant (VOC-202012/01) that was predominant in Europe.

## RESULTS

### Baculovirus expression and localization of SARS-COV-2 spikes in Sf9 insect cells.

**FIG 1:**
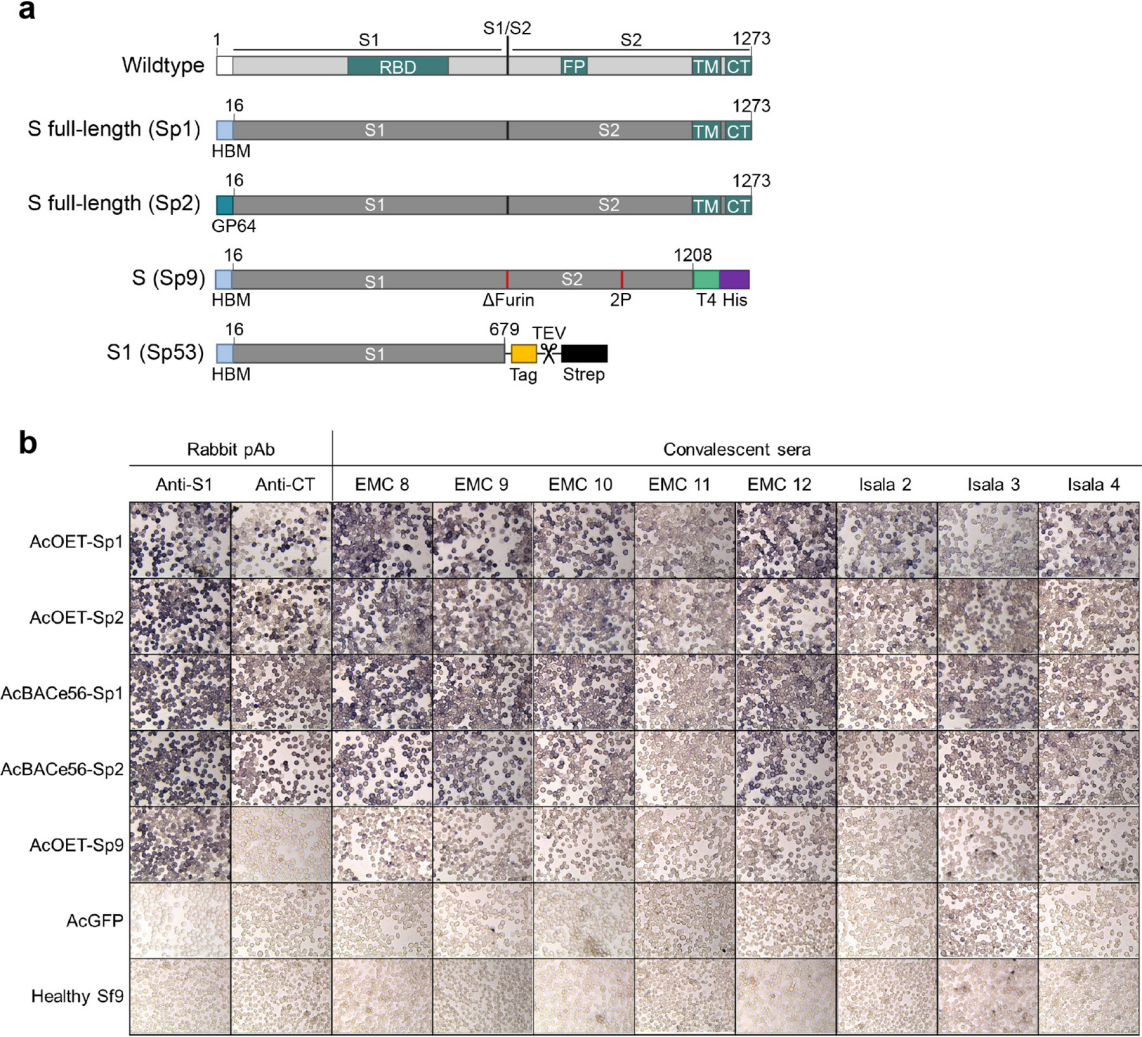
Expression of recombinant SARS-CoV-2 spike protein in insect cells. (a) In this study, recombinant baculoviruses were constructed containing spike proteins as schematically represented. (b) S full-length and S (delta TM) were detected inside Sf9 cells after staining with rabbit polyclonal sera against S1 (anti-S1) or cytoplasmic tail in S2 (anti-CT) or with human convalescent-phase sera obtained from two hospitals (EMC and Isala). pAb, polyclonal antibody.

To determine the most suitable recombinant baculovirus system to produce SARS-CoV-2 spike variants and to determine the most suitable secretion signal sequence, Sf9 cells were infected with AcBACe56 and AcOET recombinant baculoviruses encoding the SARS-CoV-2 spike (Fig. 1a). The spike proteins were similarly expressed in the cells with either the N-terminal HBM signal sequence or the baculovirus GP64 signal sequence (Fig. 1b, compare Sp1 with Sp2). Furthermore, the AcBACe56 and AcOET recombinant baculoviruses induced expression of S proteins that were recognized by convalescent-phase sera from COVID-19 patients (Fig. 1b). Baculovirus encoding GFP (AcGFP) and mock-infected Sf9 cells were included as controls and were not detected by the same sera. As expected, removal of the C-terminal transmembrane (CT) domain from the spike protein lowered the detection signal, since S was now excreted from the cells (Fig. 1b, AcOET-Sp9). Compared to AcOET, our in-house AcBACe56 produced similar yields of secreted S (see Fig. S1 in the supplemental material). Generation of recombinant AcOET baculovirus stocks demands less hands-on time than AcBACe56. Therefore, AcOET-Sp9 was used for further production and purification of secreted S.

10.1128/mBio.01813-21.2 FIG S1 Spike protein secretion. Both full spike with transmembrane domain (Sp1-2) and spike without transmembrane domain (Sp9-10) were expressed with HBM or Gp64 as a signal sequence. Recombinant baculovirus was constructed with the AcBACe56 and the AcOET virus backbones. Sf9 cells were infected with recombinant baculovirus, and protein secretion in the medium was analyzed by SDS-PAGE (stain-free) (B) followed by Western blotting with polyclonal anti-S1 serum (A). Download FIG S1, TIF file, 2.5 MB. Copyright © 2021 van Oosten et al. 2021 van Oosten et al. https://creativecommons.org/licenses/by/4.0/ This content is distributed under the terms of the Creative Commons Attribution 4.0 International license.

### Immunogenicity of a SARS-COV-2 subunit vaccine from insect cells.

**FIG 2:**
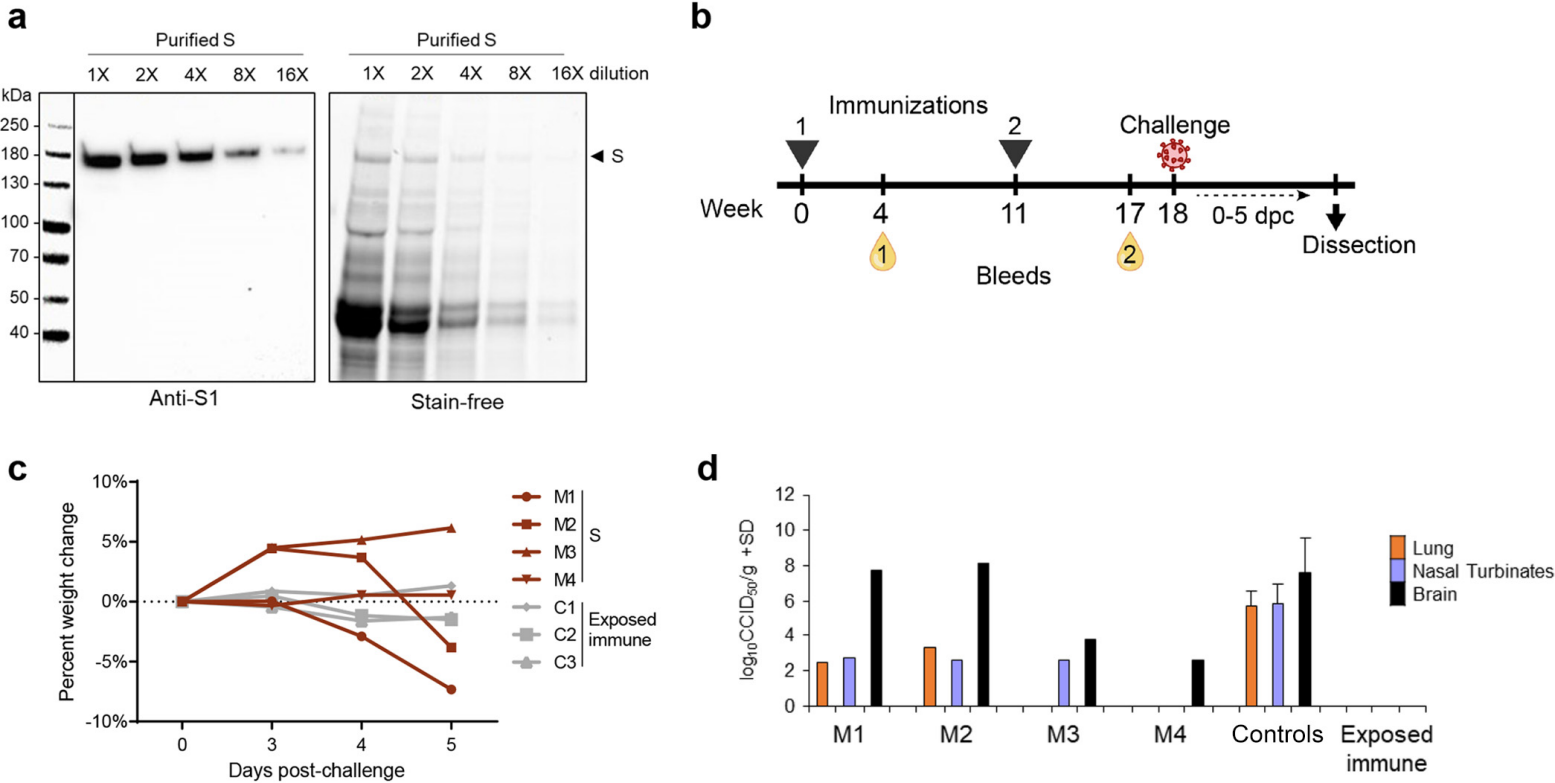
Production of S and immunization of K18-hACE2 mice with two doses of adjuvanted S induce limited nAb responses and result in partial protection from SARS-CoV-2 challenge. (a) S was produced with the ExpiSf expression system and purified from medium. The elution fractions were pooled and analyzed on SDS-PAGE (stain-free) and Western blotting (anti-S1) in a serial dilution. (b) K18-hACE2 mice (*n* = 4, M1 to M4) were immunized with two doses of 5 μg S and AS01 adjuvant, where blood was collected after each vaccination. At 18 weeks postprime, the mice were challenged intranasally with SARS-CoV-2. (c) From 0 to 5 days postchallenge (dpc), body weight change was monitored. (d) At 5 dpc, the viremia in lungs, nasal turbinates, and brain were measured. Mice vaccinated with UV-inactivated SARS-CoV-2 served as an exposed immune control (*n* = 3, C1-C3), and nonvaccinated controls (*n* = 12) were included in viremia titrations.

The prefusion-stabilized S was produced with AcOET-Sp9 baculoviruses in ExpiSf9 cells cultured in shake flasks in chemically defined medium. After purification, the recombinant protein yield was estimated to be 180 μg S protein per liter of culture fluid with relatively low purity (Fig. 2a). The insect cell proteins in the culture fluid contaminated the S protein and could not be removed with a single purification step. We subsequently used this S with AS01 adjuvant in a vaccination challenge study in four K18-hACE2 transgenic mice (Fig. 2b). After two intramuscular immunizations with 5 μg adjuvanted S, we measured virus-neutralizing activity in sera of the vaccinated mice. Two mice (M3 and M4) had detectable neutralizing antibody responses, both with a reciprocal serum dilution titer of 15. There were no measurable amounts of neutralizing antibodies in the other two animals (M1 and M2) or in the sera obtained after the first immunization. Mice were challenged with an intranasal sublethal dose of SARS-CoV-2 at 18 weeks postprime. Two mice, M1 and M2, developed disease symptoms and showed a loss of body weight, in contrast to the exposed-immune control group (Fig. 2c). At 5 days postchallenge, SARS-CoV-2 virus was found in their lungs, nasal turbinate, and brains (Fig. 2d). This demonstrates SARS-CoV-2 infection and replication in the vaccinated K18-hACE2 mice (50, 51), similar to the nonvaccinated controls (Fig. 2d). M3 and M4 were protected from clinical COVID-19 disease and weight loss similar to the exposed-immune control group (Fig. 2c). Although no SARS-CoV-2 was detected in the lungs, virus was measured in the nasal turbinate of M3 and in the brains of both M3 and M4 (Fig. 2d). As a result, two intramuscular doses of adjuvanted S could provide partial protection against SARS-CoV-2 in hACE2 mice.

### Production of strep-tagged S1 subunit increases yield and purity.

**FIG 3:**
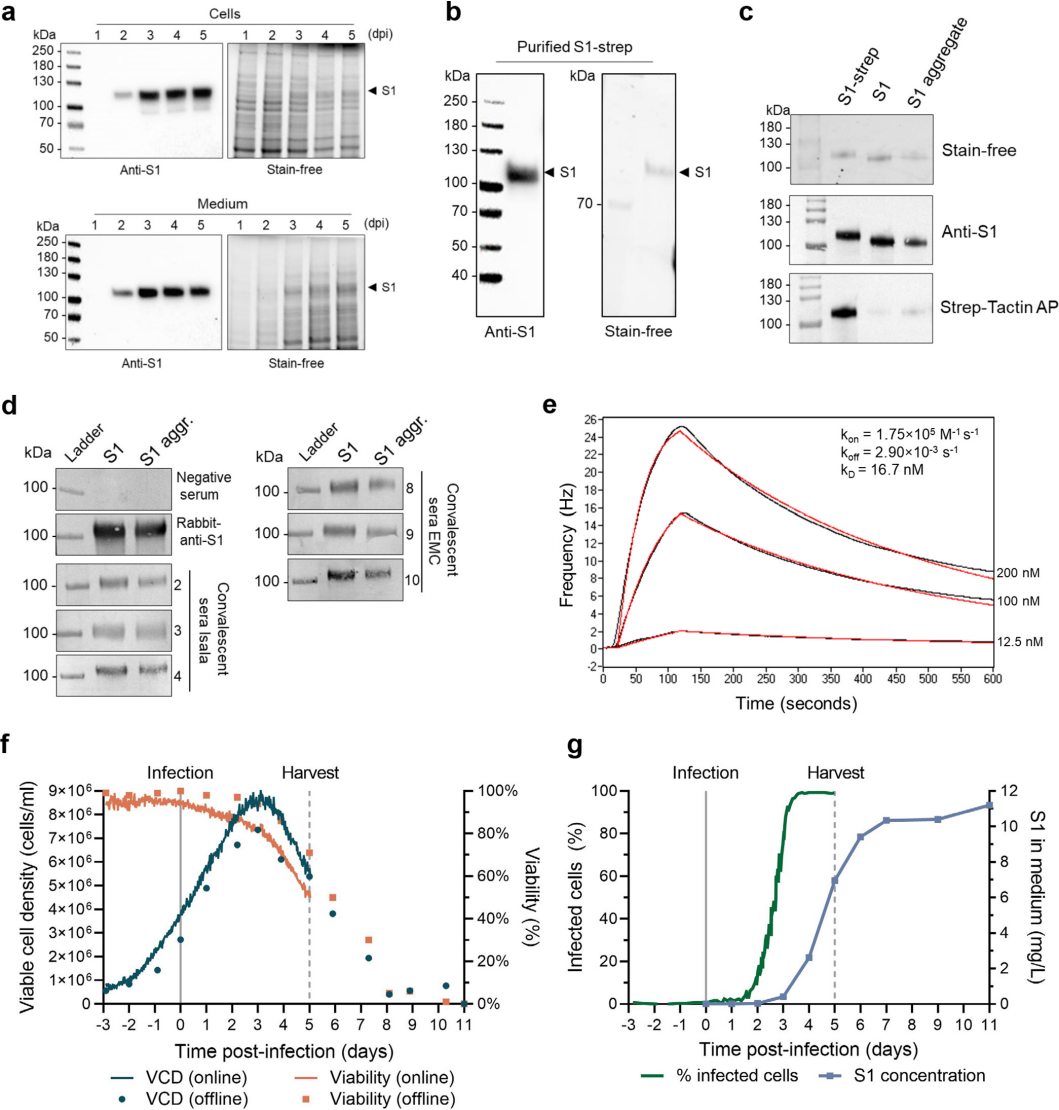
Production and analyses of recombinant S1 protein in shake flasks and bioreactor. (a) S1 was produced with the ExpiSf9 expression system in chemically defined medium and was detected in cells and culture fluid on SDS-PAGE (stain-free) and Western blot (anti-S1). (b and c) S1 was purified from the culture fluid (b) and fractions before strep-tag removal (S1-strep) and after (S1 and S1 aggregate) were analyzed (c). (d) S1 and S1 aggregate (S1 aggr.) bind to COVID-19 convalescent-phase sera from Isala Zwolle (serum numbers 2, 3, 4) or from EMC Rotterdam (serum numbers 8, 9, 10) on Western blot. COVID-19 naive serum served as a negative control (Isala 7) and rabbit-anti-S1 (Sino Biological) as a positive control. (e) S1 also binds to immobilized ACE2, where real-time binding (black curves) was fitted in a 1:1 simple binding model (red curve). (f and g) Production of S1 was scaled up from shake-flask cultures to a 3-liter bioreactor. At time −3 days, ExpiSf cells were seeded, at *t* = 0 (straight line), and the cells were infected at an MOI  of  0.01. The reactor was harvested at 5 dpi (dotted line), where 60 ml of the culture was transferred to a shake flask and continued (offline) sampling. (f) Viable cell density (VCD) and viability were monitored continuously (line), and samples were taken daily for extra offline measurements (dots). (g) Fraction of infected cells from total cells in the bioreactor and SARS-CoV-2 spike protein secreted in the culture supernatant measured by ELISA in milligrams per liter of culture.

We next aimed to produce the S1 subdomain to improve protein yield and purity after purification. Moreover, the adjuvanted spike subunit vaccine did not completely inhibit viral infection in K18-hACE2 transgenic mice. To improve the immunogenicity of the S1 antigen, the nanoparticle-based AP205 VLP display system was used. The secreted S1 (Fig. 1a, Sp53) was produced with AcOET-Sp53 baculoviruses in ExpiSf9 cells in shake flasks. SDS-PAGE and Western blot analyses showed that S1 was successfully expressed and secreted into the culture fluid (Fig. 3a) and could be purified efficiently using strep-tag affinity chromatography (Fig. 3b). The strep-tag affinity column had a higher specificity than the His-tag nickel affinity column and resulted in a relatively pure S1 protein compared to the purity of S after immobilized-metal affinity chromatography. The C-terminal strep-tag was removed by tobacco etch virus (TEV) protease cleavage, resulting in TEV-cleaved S1 (60%) and an S1 aggregate (30%) (Fig. S2). Removal of the strep-tag was demonstrated by a shift in protein size on SDS-PAGE and a loss of signal from StrepTactin-alkaline phosphatase (AP) antibody on Western blotting (Fig. 3c). Both S1 and the S1 aggregate fractions show a band with the same mass on denaturing SDS-PAGE and binding on Western blotting to rabbit-anti-S1 and several human COVID-19 convalescent-phase sera (Fig. 3d). Moreover, S1 has a high binding affinity to immobilized ACE2, with a dissociation constant (*k_D_*) of 16.7 nM (Fig. 3e), which is in a similar range as previously reported (10, 34, 52). This indicates that the receptor-binding site within S1 is exposed and available to interact with ACE2.

10.1128/mBio.01813-21.3 FIG S2 Removal of C-terminal triple strep-tag from S1 by TEV protease cleavage. The purified S1 was incubated overnight with 10:1 (wt/wt) TEV protease to S1. The cleaved product was run over a preparative Superdex S200 column, resulting in the pooled separation of S1 aggregate (peak 1, 75 to 98 ml), S1 (peak 2, 99 to 117 ml), and other proteins, including TEV protease (peak 3, 135 to 175 ml). Download FIG S2, TIF file, 2.3 MB. Copyright © 2021 van Oosten et al. 2021 van Oosten et al. https://creativecommons.org/licenses/by/4.0/ This content is distributed under the terms of the Creative Commons Attribution 4.0 International license.

The secreted S1 production was further scaled up from shake flasks to a 3-liter bioreactor system infected at low multiplicity of infection (MOI). Cells were grown for 3 days before infection with baculovirus. The reactor was monitored continuously online using a holographic microscope and was sampled daily for offline measurements. The maximum viable cell density (VCD) was monitored both online and offline and peaked at 3 dpi (Fig. 3f). At 4 dpi, all detected cells showed signs of baculovirus infection (Fig. 3g). The bioreactor was harvested at 5 dpi when viability dropped below 70%. The culture fluid then contained 7 mg/liter S1, which further increased to 11 mg/liter during subsequent shake flask cultivation (Fig. 3g). This is a substantial increase in protein yield compared to the 0.18 mg/liter that was obtained for the S protein.

### Analyses of S1 structure, folding, and glycosylation.

**FIG 4:**
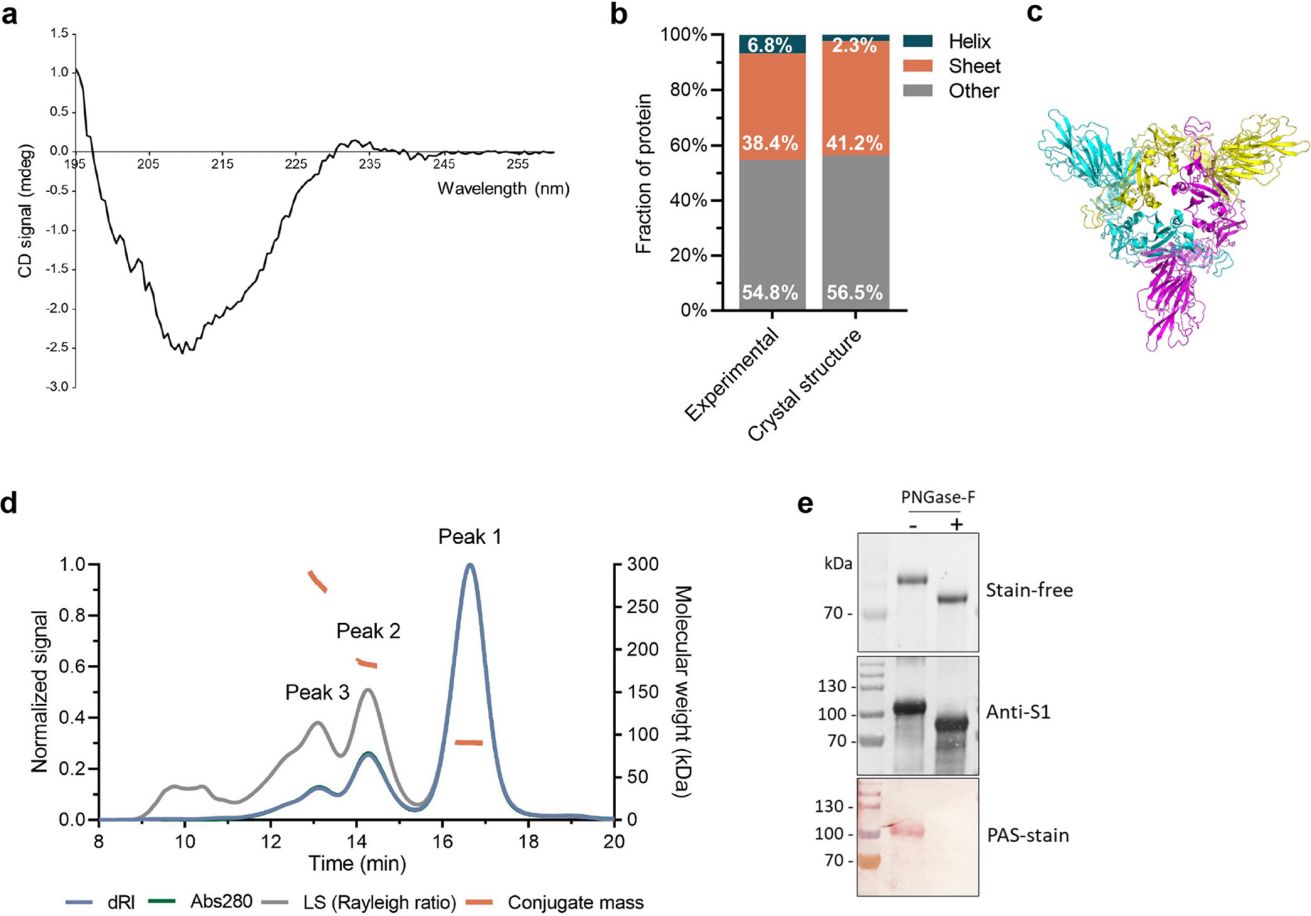
Analysis of glycosylation and oligomerization of Sf9-cell-produced S1 protein. (a) Far-UV CD spectrum of S1 at 20°C and the resulting percentage of helixes, sheets, and other structures within S1. (b) Data derived from CD measurements (experimental) were compared to those from wild-type S1 (crystal structure). (c) The proposed structure of S1 in trimeric conformation, based on the truncation of the full-length SARS-CoV-2 structure (77). (d) A representative chromatogram of S1 on a Superdex 200 increase 10/30 column, as detected by differential refractive index (dRI), absorption at 280 nm (A280), and light scattering (LS). The molecular weight of the species eluting in each indicated peak is shown in orange (conjugate mass). (e) S1 N-linked glycosylation was further confirmed by PNGase-F treatment and PAS staining. TEV-cleaved S1 was treated with PNGase-F. Treated (+) and nontreated (−) samples were analyzed on SDS-PAGE (stain-free) and Western blot (anti-S1). Glycosylation was confirmed by PAS stain.

The structure, folding, and glycosylation of insect cell-derived S1 were analyzed by several methods. The secondary structure content was calculated from the far-UV circular dichroism (CD) spectrum of S1 (Fig. 4a). With 6.8% α-helices and 38.4% β-sheets, the secondary structure content of S1 was highly similar to what was expected based on the wild-type (WT) SARS-CoV-2 spike structure (Fig. 4b and c) (i.e., 2.3% helices, 41.2% sheets). Thus, CD confirms that at 20°C the purified spike S1 domain mostly retained its structural elements.

The oligomerization state, molar mass, and glycosylation of S1 were determined by duplicate size-exclusion chromatography coupled with multiangle light scattering (SEC-MALS) experiments. Three oligomerization states could be distinguished in the chromatograms, as monitored by absorbance of UV light at 280 nm (Abs280), refractive index (dRI), and light scattering (LS) (Fig. 4d, Fig. S3). Conjugate analysis revealed that peak 1 represented a glycosylated protein species with a molecular weight of 90.9 ± 0.2 kDa. This glycosylated protein consisted of a protein component (82.4% ± 1.1%) with a molecular weight of 74.9 ± 1.0 kDa and a glycan component (17.6% ± 1.1%) with a molecular weight of 16.0 ± 0.2 kDa. The molecular weight of the protein component in peak 1 is in good agreement with the 77.1 kDa that is expected for the S1 protein monomer based on the amino acid sequence. The d*n*/d*c* value (i.e., the refractive index increment) for the conjugate (i.e., 0.1775 ml/g) was obtained and was used to calculate the molecular weights of the species in elution peaks 2 and 3. The protein species were found to be 183.4 ± 0.4 kDa for peak 2 and 279.8 ± 1.6 kDa for peak 3. Thus, the protein species eluting in peaks 1, 2, and 3 corresponded to an S1 monomer, dimer, and trimer, respectively. Under these conditions, the monomer is clearly the predominant S1 form.

10.1128/mBio.01813-21.4 FIG S3 Duplicate run SEC-MALS of S1. Chromatogram of S1 on a Superdex 200 increase 10/30 column, as detected by differential refractive index (dRI), absorption at 280 nm (A280), and light scattering (LS). The molecular weight of the species eluting in each indicated peak is shown in orange. Download FIG S3, TIF file, 3.0 MB. Copyright © 2021 van Oosten et al. 2021 van Oosten et al. https://creativecommons.org/licenses/by/4.0/ This content is distributed under the terms of the Creative Commons Attribution 4.0 International license.

Glycosylation of S1 was further confirmed by PNGase-F treatment and Periodic acid-Schiff (PAS) staining of the protein. The PNGase-F treatment, which removes N-linked glycans, caused a shift in protein migration on SDS-PAGE gel and a loss of PAS staining of the Western blot membrane (Fig. 4e). Together, they substantiate the results obtained with SEC-MALS. Thus, S1 produced in Sf9 cells is heavily glycosylated, akin to SARS-CoV-2 proteins that were produced in HEK293 cells (53).

### Display of S1 on AP205 VLPs.

**FIG 5:**
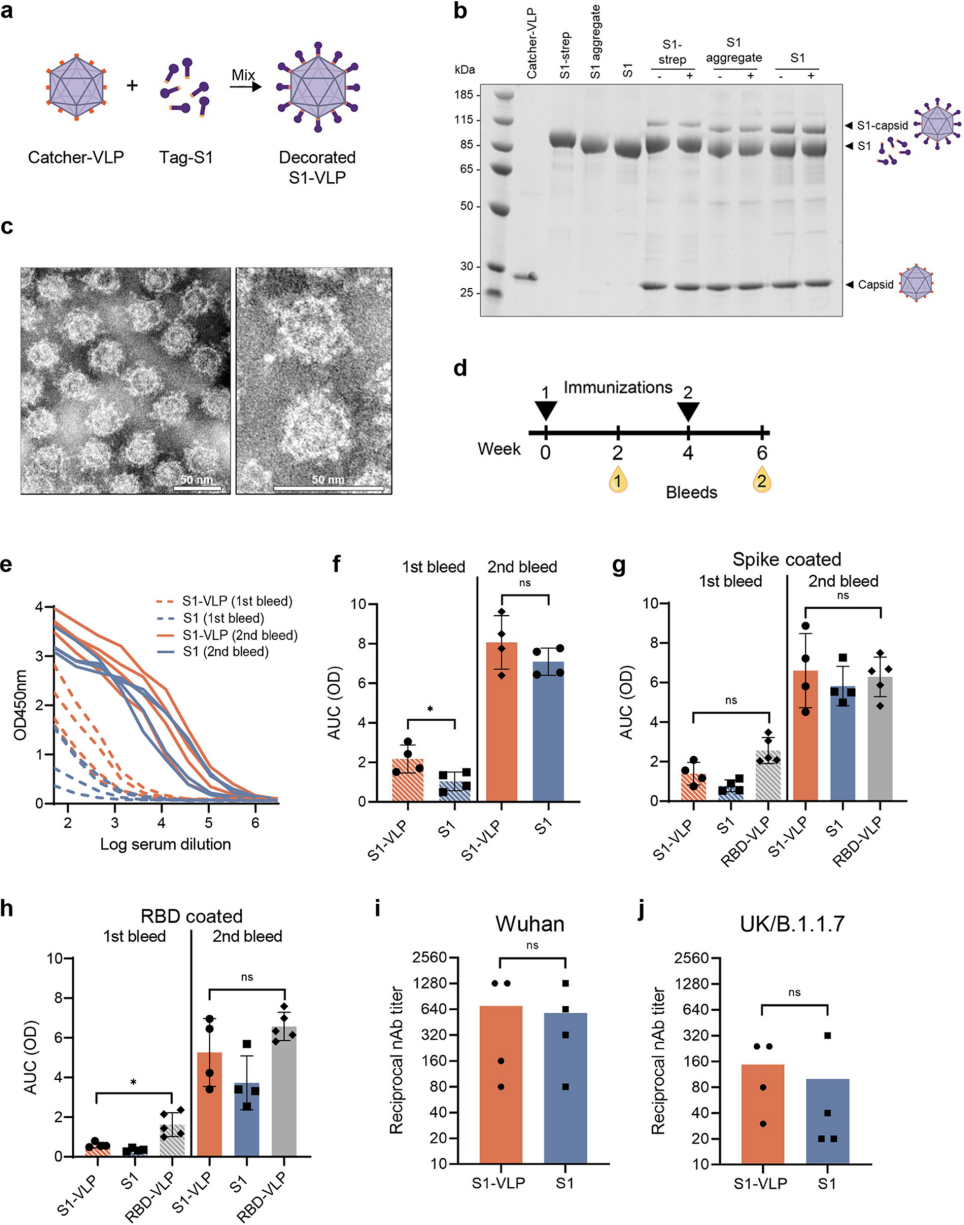
Low-dose vaccination of BALB/c mice with soluble S1 and S1-VLP induces potent neutralizing antibody responses. (a) S1 was coupled to VLPs by a coupling reaction as schematically represented. The catcher-VLPs are mixed with tag-S1, which results in a covalent display (decorated S1-VLP) that is visible on a reduced, denaturing SDS-PAGE gel. The individual components (catcher-VLP and S1) are shown. (b) Antigens were mixed with VLP and were analyzed again before (−) and after (+) a centrifugation step. An extra band appears representing the covalently fused S1 to a single AP205 capsid (S1-capsid). (c) The VLPs were purified from the uncoupled S1 subunits and visualized by negative-stain transmission electron microscopy (TEM). The scale bar represents 50 nm. (d) BALB/c mice (*n* = 4 per group) were vaccinated with 0.5 μg soluble S1 or 0.5 μg S1-VLP with Addavax adjuvant. Blood was collected 2 weeks postprime (1st bleed) and 2 weeks postboost (2nd bleed). (e) IgG antibodies against SARS-CoV-2 spike were analyzed in a dilution series in an ELISA. (f to h) The IgG titers were expressed as area under the curve (AUC) values. (f) The IgG titers in plates coated with SARS-CoV-2 spike were compared for mice vaccinated with S1 or S1-VLP. (h and g) Sera were measured on ELISA plates coated with RBD (g) or coated with full spike (h) and were compared to sera of mice vaccinated twice with 5 μg RBD-VLP. (i and j) The reciprocal neutralizing antibody titers after the second vaccination are shown for neutralizing SARS-CoV-2 Wuhan (L008) (i) or UK/B.1.1.7 (j) strain. A two-sided nonparametric Mann-Whitney *t* test was used for statistical comparison. ns, nonsignificant; *, *P* < 0.05. Significance is marked with an asterisk in panels e (*P* = 0.0286) and g (*P* = 0.0159).

To improve the immunogenicity of the prototype vaccine, the S1 and S1 aggregate fractions were individually mixed in equimolar ratios with catcher-VLPs in an overnight coupling reaction (Fig. 5a). We included the S1 protein that still contained the C-terminal strep-tag (S1-strep) as a control. The coupling efficiency was assessed on a reducing SDS-PAGE as shown in Fig. 5b. An additional band of higher molecular mass appeared on the SDS-PAGE gel, corresponding to one S1 molecule covalently bound to one AP205 capsid. Removal of the C-terminal strep-tag by TEV protease cleavage improved the coupling efficiency. In that case, the coupling efficiency was estimated as at least 10%. This suggests that, on average, each VLP displayed at least 18 S1 proteins on its surface. A centrifugation step after coupling (stability spin test) revealed that the coupling was stable and the S1-VLP was not prone to aggregation. These results are similar to what was seen for a previously developed RBD-VLP vaccine (34). After purification of the VLPs from the remaining soluble S1, the VLPs are intact and of good quality, and the S1 domains are uniformly distributed among the VLP particles, as seen by transmission electron microscopy (TEM) (Fig. 5c).

### Immunogenicity of the S1-VLP nanoparticle vaccine.

The immunogenicity of the S1-VLP was assessed in a mouse immunization study and was compared to mice vaccinated with soluble S1 protein. Before vaccine formulation of S1-VLP, the unbound S1 was removed from the coupling reaction mix by using ultracentrifugation. BALB/c mice (*n* = 4) received two intramuscular immunizations containing only 0.5 μg S1-VLP or 0.5 μg S1 per dose, formulated with Addavax, and blood was collected 2 weeks after each vaccination (Fig. 5d). All mice showed potent immune responses, especially after the second immunization, as measured in an IgG protein enzyme-linked immunosorbent assay (ELISA) (Fig. 5e). The areas under the curve (AUC) were derived from the ELISA data and showed a slight difference in IgG levels after the first immunization between the two groups, with the S1-VLP vaccine performing better (Fig. 5f). After the second immunization, however, the effect of the VLP coupling was less clear, possibly because in both groups an adjuvant was used.

These sera were compared to sera of mice vaccinated with 5 μg of the previously developed RBD-VLP vaccine (34). Even though the antigen concentration in the S1-VLP vaccine trial was 10-fold lower, all groups had comparably high antibody responses to the spike protein, as determined by ELISA (Fig. 5g). The IgG titers specific for the SARS-CoV-2 RBD domain were higher in the RBD-VLP vaccinated mice than our S1- and S1-VLP-vaccinated mice after the first bleed but not after the second bleed (Fig. 5h).

Finally, after the second vaccination, all sera contained substantial virus-neutralizing antibody titers against the Wuhan (L008) SARS-CoV-2 strain, on which the vaccine was based (Fig. 5i). Importantly, sera were also able to neutralize the UK (B.1.1.7) strain of SARS-CoV-2 (Fig. 5j).

## DISCUSSION

The development and/or improvement of effective SARS-CoV-2 vaccines are still a priority worldwide. Protein-based vaccines are needed to vaccinate the world population and to boost immunity against emerging variants. These vaccines are known for their inherent long-term safety and efficacy after administration and lack of preexisting antivector immunity (54). Moreover, the production safety, production costs, and vaccine storage temperatures are advantageous compared to mRNA and adenovirus vector vaccines, especially for scale-up manufacturing and global distribution (55). In this study, we have used the versatile and scalable baculovirus expression vector system to generate a two-component nanoparticle vaccine that induced a potent neutralizing antibody response against SARS-CoV-2 variants.

Both secreted S and S1 were produced with the ExpiSf9 expression system in chemically defined medium, which is both serum-free and protein-free. A yield after purification of 0.18 mg/liter for S was obtained. This is relatively low, although production yields of prefusion-stabilized S vary greatly between expression systems. Yields of 0.5 mg/liter S have been reported for FreeStyle 293 cells (10), 5 mg/liter for HEK293 cells (56), and up to 200 mg/liter for stably transfected CHO cells (57). Whereas trimeric S is often unstable (58, 59), smaller spike protein domains, for example, S1 or RBD, are generally more stable and result in higher yields. RBD is produced up to 50 to 60 mg/liter in Schneider-2 insect cell lines and Expi239F cells, respectively (34, 43), but production yields of the S1 subunit have not been reported to our knowledge. During our scale-up of S1 production in a stirred-tank bioreactor, we measured a maximum yield of 11 mg/liter culture, which might be further increased by subsequent parameter optimization that could be facilitated by online monitoring of the baculovirus infection process. The clear advantages of baculovirus expression over other expression platforms are its excellent safety profile, the rapid scalability to bioreactors up to several thousand liters, and its demonstrated use in the production of protein and VLP-based human vaccines (18, 19, 30). Based on the dosage of SARS-CoV-2 subunit vaccines in current phase 3 clinical trials (5 μg/dose, Novavax, Clinical Trials registration no. NCT04583995; 10 μg/dose, Sanofi Pasteur, Clinical Trials registration no. NCT04904549) and a licensed subunit vaccine made with the BEVS (135 to 180 μg/dose; FluBlok [60]), a million-dose vaccine batch of S1-VLP theoretically can be produced in a single insect cell bioreactor. Moreover, as demonstrated for the Flublok influenza vaccine, the production process can easily be adapted to new protein variants in only 6 weeks (30, 61).

Using multiple analyses, we have demonstrated correct folding, ACE2 affinity, and extensive glycosylation of the insect cell-derived S1. While glycosylation patterns in insect cells are less complex and more homogeneous than in mammalian expression systems (53, 62), there are no indications that insect glycosylation patterns positively or negatively affect the immunogenicity of SARS-CoV-2 or other glycoprotein-based vaccines (13, 17, 31).

In a first attempt to investigate the immunogenicity of insect cell-derived SARS-CoV-2 antigen, adjuvanted S was tested in a vaccination-challenge experiment in K18-hACE2 transgenic mice, but only partial protection against sublethal SARS-CoV-2 challenge was obtained. The impurities in the His-tag-purified S subunit, including glycosylated insect cell proteins, lower the effective dose to induce a specific antibody response against S. This is in line with clinical studies in which low immune responses of baculovirus-expressed, adjuvanted S protein subunit in certain age groups were reported, and this may lead to a higher reactogenicity of the vaccine (35). Thus, a better SARS-CoV-2 antigen presentation was needed, and this was accomplished by coupling S1 subunits to AP205 VLPs (34).

The tag/catcher coupling of S1 to the VLPs was increased after removal of the strep-tag and was estimated to be at least 10%. Display of the SARS-CoV-2 RBD domain onto AP205 VLPs showed 33 to 45% coupling efficiency (34), and display of other proteins ranged from 22 to 88%, which negatively correlated with the size of the antigen (45). The coupling efficiency might be further improved by increasing the coupling pressure where the antigen is present in excess in the mixture. The coupling also is potentially facing partial steric hindrance between the relatively large, glycosylated S1 antigens, and it may be useful in future studies to experiment with flexible linkers at the S1 C terminus to provide additional space to further improve antigen coupling. Moreover, S1 is highly glycosylated, and these glycans might further impair efficient coupling by steric hindrance between S1 domains. We have displayed an average of 18 relatively large S1 proteins on each 30-nm VLP, which is spaced comparably to the spikes on the wild-type SARS-CoV-2 virion (52, 63, 64).

The S1-VLPs (and S1 subunit for comparison) were used to immunize BALB/c mice twice with a low dose of 0.5 μg per mouse. The vaccines were adjuvanted with AddaVax, an MF59-like oil-in-water emulsion. Comparison between adjuvants in SARS-CoV-2 subunit vaccines has shown that these squalene-based oil-in-water emulsions are among the most potent adjuvants (16, 44, 65). An adjuvanting effect of the VLP was observed only after the first immunization, where a significant difference between the S1 subunit and S1-VLP was observed in the amount of IgG produced. After the second immunization, the S1 subunit and S1-VLP performed similarly in inducing potent immune responses. The boosting effect of the second immunization and the addition of Addavax adjuvant in both vaccine groups might be obscuring the intrinsic adjuvanting effect of the AP205 VLP (42).

An RBD-VLP prototype vaccine is currently under evaluation in the COUGH-1 clinical phase I/II trial (National Library of Medicine, Clinical Trials registration no. NCT04839146) and makes use of the same AP205 VLPs but with an RBD antigen expressed in S2 *Drosophila* cells (34). We show that our S1-VLP vaccine reaches IgG titers comparable to those of the RBD-VLP but with a 10-times-lower antigen dose (0.5 μg for S1-VLP versus 5 μg for RBD-VLP). In line with this result, a comparison of HEK293-cell-derived S1 and RBD subunits showed that S1 gave superior immune responses when formulated as a subunit vaccine (33). It remains to be seen if enhanced immunogenicity of S1 compared to RBD can compensate for the less efficient S1-VLP coupling. In our study, both vaccines appear equally potent in generating SARS-CoV-2-specific antibodies.

Neutralizing antibodies are an important immune correlate of protection (6, 66, 67); therefore, it was an important finding that all mice generated high neutralizing antibody titers for both the Wuhan L008 as well as the B.1.1.7 UK variant of SARS-CoV-2. The neutralizing antibody titer determinations have been performed in separate assays and, thus, cannot be directly compared, but it is feasible that protection against B.1.1.7 is somewhat reduced due to the presence of several mutations in the S1 domain (HV69-70del, Y144del, N501Y, A570D, and D614G). With RBD-based vaccines, this difference would only be a single amino acid (N501Y) (68). Immunogenicity against current and emerging variants might be further improved by the development of a multivalent vaccine, i.e., a VLP displaying a mixture of S1 variants. This can result in a more focused immune response toward conserved protein domains and broader protection against variants, as was shown for HIV (69). Neutralizing antibody responses against other SARS-CoV-2 variants of concern, like B.1.1.7, are important for future vaccine designs and for the progression of vaccine candidates through clinical trials toward licensing (70). With novel SARS-CoV-2 variants emerging in the years to come, these two-component nanoparticle vaccines can be quickly adapted as booster vaccines by simply updating the antigen component.

## MATERIALS AND METHODS

### Insect cells.

Spodoptera frugiperda Sf9 insect cells were cultured in Sf900II serum-free medium (Gibco, Thermo Fisher) with 50 μg/ml gentamicin. ExpiSf9 cells were cultured in ExpiSf chemically defined medium (CDM) (Thermo Fisher). Both cell lines were cultured in suspension in shaker flasks at 27°C and 110 rpm in an orbital shaker.

### Plasmid design and construction.

All constructs are based on the SARS-CoV-2 Wuhan isolate (accession no. QIA20044.1) spike gene that was codon optimized for production in Drosophila melanogaster (34). For endoplasmic reticulum (ER) translocation and secretion, the native N-terminal signal sequence was replaced with either the Honeybee Melittin (HBM) or AcMNPV GP64 signal sequence. PCR was performed on either the wild-type spike sequence or the prefusion-stabilized sequence with primers found in Table S1 in the supplemental material (Q5 high-fidelity 2× master mix; New England BioLabs). All PCR products were flanked by Gateway AttB sites. The PCR products were gel purified with the Illustra GFX gel band purification kit (GE-Healthcare) and were then gateway cloned into the pDONR207 (Thermo Fisher), pDest8 (Thermo Fisher), and/or the pOET1 Gateway vectors (Oxford Expression Technologies) according to the protocol of Gateway cloning (Invitrogen).

10.1128/mBio.01813-21.1 TABLE S1 Primers used in this study for amplification of spike sequences and gateway cloning into pDONR207. Download Table S1, DOCX file, 0.01 MB. Copyright © 2021 van Oosten et al. 2021 van Oosten et al. https://creativecommons.org/licenses/by/4.0/ This content is distributed under the terms of the Creative Commons Attribution 4.0 International license.

Multiple constructs were designed based on the full-length spike (S full length), the prefusion stabilized secreted spike (S), and the S1 domain (Fig. 1a). First, the S full-length spike constructs contain the wild-type spike coding sequence (residues 16 to 1237) with an N-terminal HBM signal sequence (Sp1) or GP64 signal sequence (Sp2). Second, the S spike construct encodes an N-terminal HBM signal sequence, the prefusion stabilized ectodomain (residues 16 to 1208), a C-terminal T4 fibritin trimerization signal (T4 foldon), and an 8× histidine tag (His) (Sp9). Third, the S1 domain (residues 16 to 679) was PCR amplified and cloned into the pDONR207 vector. At the C terminus of the S1 gene in pDONR207, a synthetic DNA fragment (IDT DNA) was inserted by SapI restriction cloning (New England BioLabs). The resulting construct (Sp53) encodes the S1 domain with a C-terminal GSGSGS linker, the proprietary split-protein tag, tobacco etch virus (TEV) protease cleavage site (ENLYFQS), and triple strep-tag (WSHPQFEKGGGSGGGSGGSAWSHPQFEKGGGSGGGSGGSAWSHPQFEK).

### Recombinant baculoviruses.

Recombinant baculoviruses of Autographa californica multicapsid nucleopolyhedrovirus (AcMNPV) were generated in two ways. First, pDest8 plasmids were transposed into E. coli cells containing bacmid AcBACe56 (Δcc) (71) with the bac-to-bac baculovirus expression system (Invitrogen, Thermo Fisher), followed by transfection of the bacmid into insect cells with Expres2TR transfection reagent (Expres2ion Biotechnologies). Second, pOET1 plasmids were transfected into insect cells with the baculovirus genome AcOET (Δcc Δp10 Δp74 Δp26) with the *flash*BAC Ultra kit (Oxford Expression Technologies). Baculovirus stocks were amplified in ExpiSf9 cells, and viral titers were determined by endpoint dilution assay on Sf9-easy titer cells (72). Viral titers are defined as 50% tissue culture infectious dose (TCID_50_) units per ml.

### IAPMA.

Production of S full-length in insect cells was assessed by immunoalkaline phosphatase monolayer assay (IAPMA). Sf9 cells were infected with recombinant baculovirus at a multiplicity of infection (MOI) of 1 TCID_50_ per cell. At 3 days postinfection, cells were washed once with phosphate-buffered saline (PBS) and fixed in acetone-ethanol (1:1) fixative, followed by another wash step. Cells were incubated for 30 min at 37°C with one of the following primary antibodies: rabbit-anti-S1 (40591-T62; Sino Biological; binds the SARS-CoV-2 S1 domain), rabbit-anti-S2 (ab272504; Abcam; binds the cytoplasmic tail [CT] of S2), or human convalescent-phase sera (Isala Ziekenhuis, Zwolle; Erasmus Medical Centre, Rotterdam; obtained from COVID-19 patients). All primary antisera were diluted in PBS with 0.05% Tween 20 (Merck) and 5% skim milk powder (Campina, The Netherlands). Wells were washed once with PBS and incubated with alkaline phosphatase (AP)-conjugated goat-anti-rabbit or goat-anti-human secondary antibodies (Sigma). Proteins were detected by nitrotetrazolium blue–5-bromo-4-chloro-3-indolylphosphate (NBT-BCIP) staining (Roche Diagnostics GmbH, Basel, Switzerland), and cells were visualized by using a Zeiss AxioObserver Z1m inverted microscope.

### Protein production in shake flasks.

Recombinant proteins were produced with the ExpiSf expression system (Thermo Scientific). ExpiSf9 cells were grown in 1-liter shake flasks with 250 ml cell culture volume and were diluted to a density of 5 × 10^6^ cells/ml in medium supplemented with ExpiSf enhancer. At 18 to 24 h after dilution, the cells were infected with recombinant baculovirus at an MOI of 4 to 5 TCID_50_ units per cell. The flasks were incubated for 2 to 4 days until the viability was reduced to 70%. Next, phenylmethylsulfonyl fluoride (PMSF) protease inhibitor (Roche) was added at a final concentration of 170 μg/ml. Cells were removed by centrifugation, and the pH of the resulting supernatant was increased to pH 7.8 by adding 0.1 M NaOH. The clarified supernatant was immediately used for purification or was stored at −80°C after the addition of glycerol (final concentration, 20%).

### Protein production in insect-cell bioreactor.

A 3-liter bioreactor (Applikon Biotechnology) with a 2-liter working volume was inoculated with 0.5 × 10^6^ ExpiSf9 cells/ml at 99% viability. Cells were grown in CDM at 27°C with an agitation speed of 266 rpm. The dissolved oxygen (DO) was controlled at 30% air saturation by adding pure oxygen through a macroporous L-sparger. In addition, a constant headspace flow of 0.01-vvm air was applied. Cells were infected at an MOI of 0.01 at 3 days postinoculation at a VCD between 2.0  × 10^6^ and 4.0 × 10^6^ cells/ml. The reactor was harvested at 5 days postinfection (dpi) at a cell viability around 70% as described above. The reactor was monitored online continuously and was sampled daily for offline measurements. Sixty milliliters of cell culture was transferred to a shake flask during harvesting and was sampled for another 6 days. The VCD, viability, and fraction of infected cells were measured online by differential digital holographic microscopy (DDHM) with the iLine F holographic microscope (OVIZIO). The bioreactor samples were analyzed offline for VCD and viability by trypan blue exclusion by using a TC20 automatic cell counter (Bio-Rad). The SARS-CoV-2 spike S1 concentration in the medium was determined by protein ELISA (CBK4154; AssayGenie).

### Protein purification.

His-tagged S spike protein was purified from the clarified medium by using immobilized metal ion affinity chromatography (IMAC). The expression medium was clarified by centrifugation (12,000 × *g*, 4°C). The clarified medium was loaded onto a Ni Sepharose excel (Cytiva) column that was equilibrated in equilibration buffer (20 mM Tris-Cl, 400 mM NaCl, pH 7.8). Non- and weakly bound contaminants were removed by washing with equilibration buffer. The bound proteins were eluted from the column by a 0 to 360 mM imidazole gradient in equilibration buffer. Eluted fractions were pooled and concentrated by using an Amicon Ultra-15 centrifugal filter unit (30 kDa; Merck). The concentrate was further purified by size-exclusion chromatography (SEC) with a Superdex S200 (Cytiva) column equilibrated with PBS buffer at pH 7.4. Eluted fractions were analyzed on SDS-PAGE. The fractions containing the S protein were pooled, concentrated, flash-frozen, and stored at −20°C.

Strep-tagged S1 spike protein was purified on a Strep-TactinXT superflow 5-ml cartridge (IBA GmbH). The clarified medium was treated with 15 μl/ml BioLock biotin blocking solution (IBA) to remove free biotin. It was then loaded onto the Strep-Tactin column equilibrated in 100 mM Tris-HCl, 150 mM NaCl, and 1 mM EDTA, pH 8.0 (buffer W). The column was washed with buffer W, and proteins were eluted from the column in buffer W containing 50 mM biotin. To remove the triple strep-tag, we pooled the eluted fractions and incubated them overnight at 4°C with TEV protease at a 10:1 (wt/wt) ratio in 50 mM Tris-Cl, 0.5 mM EDTA, and 1 mM dithiothreitol (DTT), pH 8.0. The protein was concentrated and further purified by using SEC as described above. Eluted fractions were analyzed with SDS-PAGE. The fractions containing S1 were pooled, flash-frozen, and stored at −20°C. Removal of the strep-tag by TEV cleavage was confirmed on Western blot immunodetection by Strep-Tactin AP-conjugated antibody (IBA Lifesciences).

### SDS-PAGE and Western blot immunodetection.

Protein samples were incubated in Laemmli buffer containing 5% β-mercaptoethanol for 10 min at 95°C and were separated on a 7.5% SDS-PAGE gel (Bio-Rad) or a 4 to 20% or 4 to 15% stain-free SDS-PAGE gel (containing photoactivatable trihalo compound; Bio-Rad). Proteins were visualized by 2.5-min UV-activated stain-free imaging (Bio-Rad GelDoc). The proteins in the gel then were blotted on a polyvinylidene difluoride (PVDF) or nitrocellulose membrane (Thermo Scientific, Bio-Rad). The membranes were blocked in PBS with 0.1% Tween 20 (PBST) supplemented with 3% skim milk powder (Campina, The Netherlands), and recombinant spike proteins were detected by immunodetection. As primary antibodies, we used rabbit-anti-S1 polyclonal serum diluted 1:3,000-1:5,000 (40591-T62; Sino Biological) or human COVID-19 convalescent-phase sera diluted 1:500 (Isala Ziekenhuis, Zwolle) or 1:1,000 (Erasmus Medical Centre, Rotterdam).

Detection was performed with either AP-conjugated secondary antibody or horseradish peroxidase (HRP)-conjugated secondary antibody diluted 1:2,500 in PBST. The AP-conjugated goat-anti-rabbit (Aligent) and AP-conjugated goat-anti-human (Sigma-Aldrich) were detected by conversion of NBT-BCIP staining (Roche Diagnostics GmbH, Basel, Switzerland). HRP-conjugated goat-anti-rabbit (Sanbio) was visualized by Clarity Western ECL substrate (Bio-Rad) by using a Chemidoc MP (Bio-Rad). The strep-tag was detected by immunodetection with Strep-Tactin AP according to the manufacturer’s protocol (2-1503-001; IBA Lifesciences).

### PNGase treatment and PAS stain.

The glycosylation status of recombinant spike proteins was analyzed by a PNGase treatment and PAS staining. Protein samples were incubated with PNGase-F (P0704; New England Biolabs) for 1 h at 37°C under denaturing conditions as described by the manufacturer. PNGase-F-treated and nontreated samples were analyzed by SDS-PAGE and Western blotting. Next, protein glycosylation was confirmed by PAS staining of the Western blot membrane. The PVDF membrane was soaked in PAS solution (1% periodic acid in 3% acetic acid) for 15 min. The membrane was washed twice with water and incubated for 15 min with Schiff’s reagent (Sigma-Aldrich). The membrane was washed once in 0.5%, wt/vol, sodium bisulfite for 5 min and rinsed with demineralized water before imaging.

### Far-UV CD spectroscopy.

The structural features of the insect cell-produced S1 were assessed by far-UV circular dichroism (CD). Purified S1 was diluted to 1.3 μM in PBS. The far-UV CD was measured by using a Jasco J715 spectropolarimeter at 20°C with 1-mm quartz cuvettes. Spectra were obtained by averaging 30 scans and were background corrected by subtracting the far-UV CD spectrum of 30 scans of PBS acquired under identical conditions. We used the CD data as the input for the BeStSel webserver (73, 74) to acquire the secondary structure content of S1. This was compared to the structural data of the S1 domain (derived from the wild-type spike protein structure) we obtained by the program STRIDE (75). In both algorithms, it was assumed that glycosylation and other protein modifications do not influence the amount of estimated secondary structures.

### SEC-MALS.

The molar mass, oligomerization state, and degree of glycosylation of S1 were determined by SEC-MALS. The S1 protein from the scaled-up production run was purified by Strep-Tactin purification followed by removal of the strep-tag by TEV protease incubation and SEC. After SEC, the second peak on the chromatogram (Fig. S2, elution 99 to 117 ml) was selected for SEC-MALS analyses to exclude large aggregates. An Infinity 1260 II high-performance liquid chromatography system (Agilent) was coupled to an Optilab dRI detector and miniDawn MALS detector (Wyatt Technologies, USA). The column thermostat and autosampler were both set to 20°C. The protein was diluted to 0.83 mg/ml in PBS (Sigma-Aldrich) and was then resolved in duplicate experiments on a Superdex 200 increase 10/30 GL column (GE Life Sciences) equilibrated in PBS. With ASTRA software (Wyatt Technologies), the absorption at 280 nm, light scattering, and refractive index properties of the eluates were collected and analyzed. The contribution of protein and glycosylation to the eluting species was determined by the conjugate analysis method in ASTRA using d*n*/d*c* values of 0.185 ml/g and 0.14 ml/g for protein and glycans, respectively, and an extinction coefficient for the protein at 280 nm of 1.2 ml/(mg·cm). The d*n*/d*c* value obtained for the conjugate was used for further molecular weight determination.

### Ace2 binding kinetics.

Interactions of S1 antigens with hAce2 protein were performed with a QCM Attana A200 Biosensor (Attana AB) as described elsewhere (34). Briefly, ExpreS^2^-expressed hACE2 at 50 μg/ml (Expres2ion Biotechnologies) was immobilized on an LNB carboxyl chip. The binding of S1 proteins to the chip was measured in a 2-fold serial dilution series (200 nM to 6.25 nM) in PBS, pH 7.4.

### Vaccinations at Queensland Institute of Medical Research Berghofer.

Cytokeratin-18 promoter (K18)-hACE2 mice were housed at Queensland Institute of Medical Research Berghofer. K18 mice (*n* = 4) received two intramuscular administrations of 50 μl vaccine, containing 25 μl purified S and 25 μl AS01 adjuvant. Blood was collected and tested for neutralizing antibodies against SARS-CoV-2 in a virus neutralization assay. The sera were diluted 1:10, and then 2-fold serial dilutions were incubated with 100 TCID_50_ of SARS-CoV-2 (strain hCoV-19/Australia/QLD02/2020) for 2 h. Samples were added to Vero E6 cells in 96-well plates, and viral cytopathic effect (CPE) was quantified on day 4 by crystal violet staining. Mice vaccinated with UV-inactivated SARS-CoV-2 provided positive-control sera, and naive mice provided negative-control sera. At 18 weeks postprime, the mice were challenged intranasally with 5 × 10^4^ TCID_50_ SARS-CoV-2 per mouse (hCoV-19/Australia/QLD02/2020). Weight change was monitored for 5 days, and viral titers were determined in the lungs, brain, and nasal turbinate.

### Coupling S1 antigen to virus-like particles.

Catcher-VLPs based on the AP205 coat protein (gene ID 956335) fused to the proprietary catcher sequence were produced in E. coli (45). The purified S1 was coupled to the catcher-VLPs and formulated as an adjuvanted vaccine as previously described (34). In short, S1 and catcher-VLPs were mixed in a 1:1 molar ratio in PBS overnight at room temperature. A part of the mix was spun down at 16,000 × *g* for 2 min to assess the stability of the coupled protein. Equal amounts of pre- and postspin samples were mixed with DTT and were heated prior to analyses by SDS-PAGE. The coupling efficiency was calculated as the percentage of AP205 capsids that had been conjugated to an S1 domain via tag-catcher interactions (76). The protein bands on the SDS-PAGE gel were analyzed by ImagequantTL software to determine the band intensity. The band intensity of the VLP subunit before the coupling reaction was divided by the equivalent protein band after coupling and was multiplied by 100. After the coupling reaction, the S1-VLP was purified from the remaining coupling mixture. This was loaded onto an OptiPrep step gradient (23%, 29%, and 35%) (Sigma-Aldrich) and was centrifuged at 47,800 rpm for 3.30 h. Buffer exchange was then performed by dialysis in PBS.

### TEM of the S1-VLP vaccine.

The quality of the VLPs after coupling, purification, and buffer exchange was assessed by transmission electron microscopy (TEM). For this, 1 μl VLP mixture was added to a carbon-coated copper grid and was incubated for 2 min at room temperature. The grid was washed once with Milli-Q water and was negatively stained for 30 s in 2% uranyl acetate and air-dried before observation with a JEOL 1400 plus transmission electron microscope.

### Vaccinations at University of Copenhagen.

Experiments were authorized by the Danish National Animal Experiments Inspectorate (license no. 2018-15-0201-01541; Dyreforsøgstilsynet) and performed according to national guidelines. Female BALB/c AnNRj mice were vaccinated with 0.5 μg S1 (*n* = 4) or 0.5 μg S1-VLP (*n* = 4) formulated in Addavax (Invivogen). Blood was collected 2 weeks after each immunization. The serum was isolated by two centrifugation steps at 800 × *g* for 8 min at 8°C. Specific IgG titers in serum samples were measured by ELISA. For this, the plates were coated with 0.1 μg/well recombinant ExpreS^2^-produced spike protein or RBD (Expres2ion Biotechnologies). Serum samples were added at a starting dilution of 1:50 and added in 3-fold dilutions. IgG titers were determined by optical density measurements after incubation with HRP-conjugated goat anti-mouse IgG (A16072; Life Technologies). Plates were developed by using TMB X-tra substrate (4800A; Kem-En-Tec) and absorbance measurements at 450 nM. Virus-neutralizing antibody titers in the mouse sera were determined on Vero E6 cells as described elsewhere (34) with SARS-CoV-2 Wuhan (L008) or SARS-CoV-2 UK/B.1.1.7 (VOC-202012/01) at 120 TCID_50_/well.

### Data availability.

Data are provided within this paper and are available from the authors upon reasonable request.
