# Supplementary figures and images for: Two-Component Nanoparticle Vaccine Displaying Glycosylated Spike S1 Domain Induces Neutralizing Antibody Response against SARS-CoV-2 Variants
Source: mBio. 2021 Oct 12;12(5):e01813-21. doi: 10.1128/mBio.01813-21 (PMC8510518; doi:10.1128/mBio.01813-21)

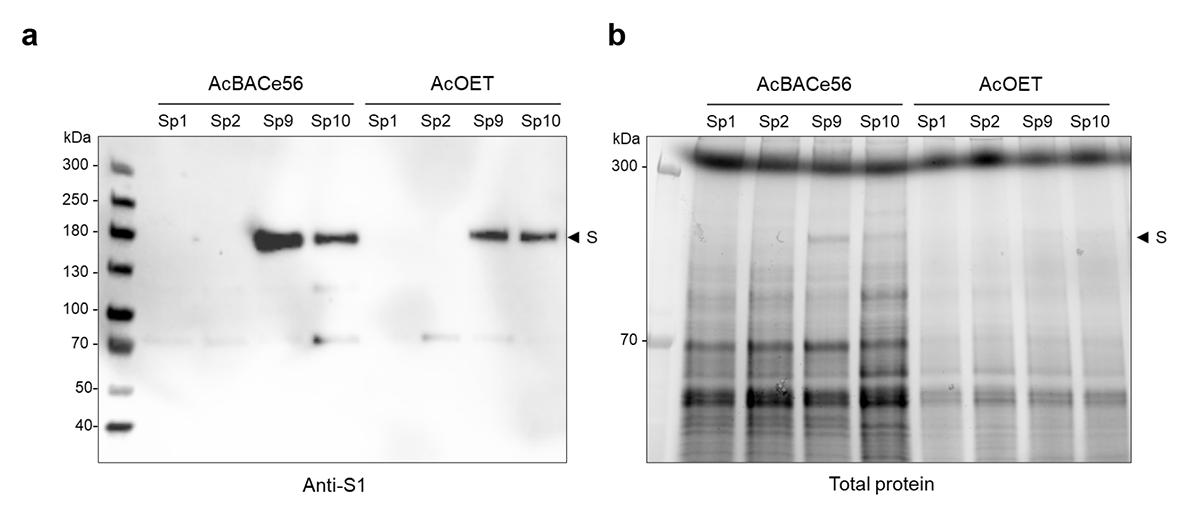

Supplement: FIG S1 [file mbio.01813-21-sf001.tif]

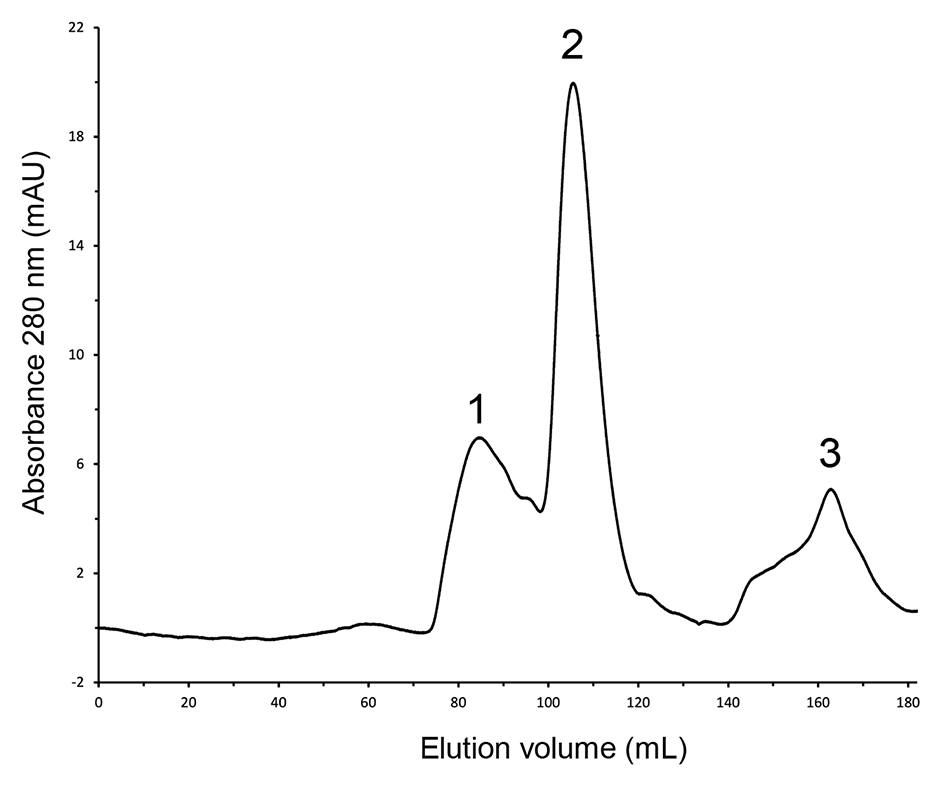

Supplement: FIG S2 [file mbio.01813-21-sf002.tif]

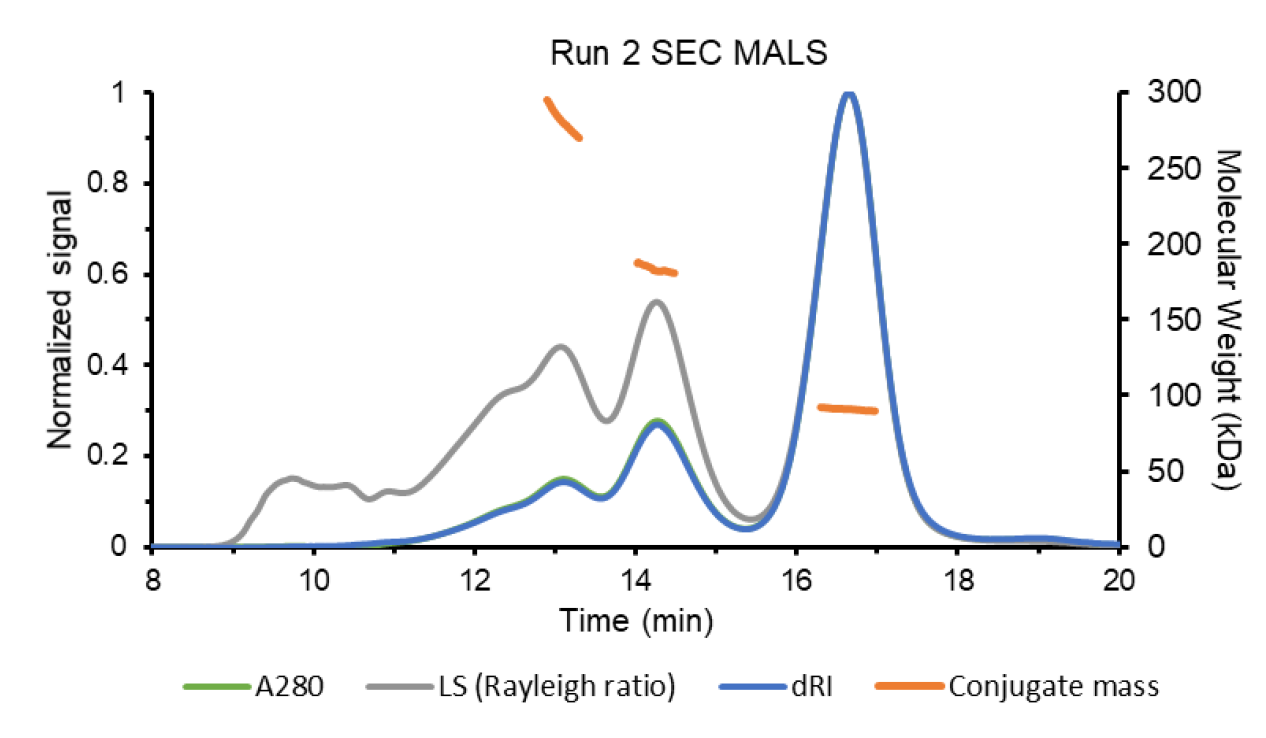

Supplement: FIG S3 [file mbio.01813-21-sf003.tif]
