# Supplementary material for: Two-Component Nanoparticle Vaccine Displaying Glycosylated Spike S1 Domain Induces Neutralizing Antibody Response against SARS-CoV-2 Variants
Source: mBio. 2021 Oct 12;12(5):e01813-21. doi: 10.1128/mBio.01813-21 (PMC8510518; doi:10.1128/mBio.01813-21)
Supplement: TABLE S1 [file mbio.01813-21-st001.docx]

**Supplementary Table 1. Primers used in this study for amplification of spike sequences and gateway cloning into pDONR207.**

| Primer name | Sequence |
| --- | --- |
| Sp1/Sp9/Sp53 forward | GGGGACAAGTTTGTACAAAAAAGCAGGCTtaGCCACCATGAAATT  CTTAGTCAACGTTGCCCTTGTTTTTATGGTCGTATACATTTCTTACA  TCTATGCCgtgaatctgaccacgcgca |
| Sp2 forward | GGGGACAAGTTTGTACAAAAAAGCAGGCTtaGCCACCATGGTAAG  CGCTATTGTTTTATATGTGCTTTTGGCGGCGGCGGCGCATTCTGCC  TTTGCGgtgaatctgaccacgcgcac |
| Sp1/Sp2 reverse | GGGGACCACTTTGTACAAGAAAGCTGGGTTgctcttcTTAcagctt  gacgcccttcagc |
| Sp9 reverse | GGGGACCACTTTGTACAAGAAAGCTGGGTAgctcttcTTAATGATG  ATGGTGATGGTGATGGTGCAGGAAGGTGGACAGCAGCACCCACTC  ACCGTCCTTGCGCACGTAAGCCTGACCGTCGCGAGGAGCCTCAGG  GATGTAACCctgctcgtatttgcccagtt |
| Sp53 reverse | GGGGACCACTTTGTACAAGAAAGCTGGGTATTAggctcttctaccg  ttcgtctgggtctgatagc |
